# Unpleasant Memories on the Web in Employment Relations: A Ricoeurian Approach

**DOI:** 10.1007/s41463-022-00138-0

**Published:** 2022-10-26

**Authors:** André Habisch, Pierre Kletz, Eva Wack

**Affiliations:** 1 Faculty of Economics and Business Administration, Catholic University of Eichstätt-Ingolstadt, Auf der Schanz 49, 85049 Ingolstadt, Germany; 2 Faculty of Management - Mandel Institute of Social Leadership, Ben-Gurion University of the Negev, P.O.B. 653, Beer-Sheva, Israel

**Keywords:** Forgetting on the web, Cybervetting, Privacy, Paul Ricoeur, Employment relations, Humanistic management

## Abstract

Cybervetting has become common practice in personnel decision-making processes of organizations. While it represents a quick and inexpensive way of obtaining additional information on employees and applicants, it gives rise to a variety of legal and ethical concerns. To limit companies’ access to personal information, a *right to be forgotten* has been introduced by the European jurisprudence. By discussing the notion of forgetting from the perspective of French hermeneutic philosopher Paul Ricoeur, the present article demonstrates that both, companies and employees, would be harmed if access to online information on applicants and current employees would be denied. Consistent with a Humanistic Management approach that promotes human dignity and flourishing in the workplace, this article proposes guidance for the responsible handling of unpleasant online memories in personnel decision-making processes, thereby following Ricoeur’s notion of forgetting as “kept in reserve”. Enabling applicants and employees to take a qualified stand on their past is more beneficial to both sides than a right to be forgotten that is questionable in several respects.

## Introduction

Advances in technology have substantially changed the role of remembering and forgetting in society. Increased competencies to save and remember everything through online media and digital storage capacity make it more convenient to remember information than to delete it, as Mayer-Schönberger (2011) notes in his book *Delete*. Human society has all along moved from the default state of “forgetting” to a state of “remembering” by externalizing memory through drawing, writing, photography, and recording (Mayer-Schönberger 2011). The shift from paper-and-ink to electronic record keeping represents a key milestone in this development (Blanchette and Johnson 2002).

In an ever-changing environment where the recent COVID-19 pandemic has forced many people to work remotely and in isolation, changes in work organization are continuously affecting the future of work and the use of new technologies in it (Bapuji et al. 2020). In particular, social media tools and access to social media content have changed decision-making processes in firms (Wade et al. 2020), challenging human resource management (HRM) in a unique way (Holland and Jeske 2017). In recruitment and selection, a breadth of externalized personal memory readily accessible on the web allows companies to conduct web searches about applicants by entering their names into search engines and reviewing their personal websites, blogs, or social media sites (Van Iddekinge et al. 2016). This practice has been referred to as “cybervetting” (Berkelaar 2017). A 2018 survey (CareerBuilder 2018) among employers reveals seven in ten employers (70 %) to use social networking sites to research job candidates during the hiring process. In existing employment relationships, social media enable employers to monitor their current employees with more opportunities and tools, even outside the workplace (Lam 2016). Thereby, nearly half of the employers (48 %) admitted to check up on current employees’ social media activities (CareerBuilder 2018).

Although cybervetting represents an easy and cost-effective way of obtaining background information for companies, applicants may be refused on the basis of online information deemed unacceptable by a potential employer (Clark and Roberts 2010). As a consequence, the use of social media information in personnel decisions raises a number of legal and ethical issues (Brown and Vaughn 2011; Jeske and Shultz 2016), whereby authors like Clark and Roberts (2010) consider cybervetting a socially irresponsible practice on the basis of privacy invasion. Once personal information is posted online, users have found it difficult to delete such information from the web due to technological and legal obstacles (Kwak et al. 2021). In order to protect users’ privacy and to limit companies’ access to personal information, the right to be forgotten was established by the European jurisprudence, offering individuals the possibility to have links to web pages containing inadequate, irrelevant, or excessive digital information removed (Kim and Kim 2017). As a means of defending users’ privacy rights, the right to be forgotten has received considerable praise. Yet, a “forced forgetting” through a right to be forgotten seems to represent an inadequate way of dealing with the issue of unpleasant information on the web (Garcia-Murillo and MacInnes 2017). The challenge is therefore not only to find adequate legal responses to exponential advancements in technology, as observed by Kwak et al. (2021), but also to adopt technology-based HRM practices that promote human dignity and flourishing. However, research on the downsides of digitalization is still in its infancy (Turel et al. 2021) and many HR practitioners seem to be ill-prepared to deal with social media in employment matters (Lam 2016). In particular, they lack clear guidelines or best practices on the use of social media in personnel decision-making (Davison et al. 2012; Landers and Schmidt 2016). With this article, we want to contribute to the existing literature by providing guidance to practitioners in making ethical personnel decisions based on information on the web.

The first aim of this paper is to provide arguments defending that a denial of access to information on the web by law is an unsuitable approach towards the promotion of human dignity and flourishing in contemporary HRM practices. By reviewing the opus of the French hermeneutic philosopher Paul Ricœur (1913–2005), we demonstrate that memories on the web cannot be erased completely through a right to be forgotten and that such a right can only provide an illusion of protection for applicants and employees. Especially in the past two decades, Ricœur’s hermeneutics approach has provided management scholars with guidance in the fields of business ethics, social responsibility, sustainable business behaviour (e.g. Dion 2017; Hummels et al. 2021), and practical wisdom in management (e.g. Habisch and Bachmann 2016). In general terms, the method of hermeneutics refers to practices of interpretation and theories of understanding, as well as associated fields of translation, communication, and information exchange (Dyer 2010). Ricœur’s hermeneutics approach, which is based on the interpretation of narrative and symbols (Reagan 1998; Dyer 2010), takes a particular interest in the analysis of human action and an understanding of the human condition (Reagan 1998). Based on his book *Memory, history, forgetting* (2004), which offers an extensive analysis of the notions of memory and forgiveness, the second aim of this paper is therefore to employ Ricœur’s (2004) concepts of forgetting as *kept in reserve* and *difficult forgiveness* to offer a Humanistic Management approach towards personnel decision-making. Following the definition of Humanistic Management by Melé (2016), such an approach “regards concern for persons and human aspects in managing organizations” (Melé 2016, p. 33). Rather than an erasure of online traces enforced by a right to be forgotten, a manager’s forgiveness of prospective and current employees’ past mistakes and appreciation for their personal learning history is more beneficial for both sides in the long run. In particular, this article contributes to the literature on Humanistic Management in HRM by showing that processes of personnel decision-making, which acknowledge and forgive individuals’ past mistakes, are better suited to foster human flourishing and personal growth than an erasure of online traces under the pressure to conform. Furthermore, humanism emphasizes human relationability and sociability, as well as harmonious cooperation to achieve a common good (Melé 2016). Hence, responsible HRM processes that allow remembering and forgiving past mistakes build trust among the members of an organization, thereby fostering constructive organizational cultures in which people can develop (Melé 2003).

The remainder of the article is structured as follows: The first part reviews the literature on the use of information from the web in personnel decisions, thereby illustrating reasons and benefits for organizations, as well as criticism relating to the ethicality of this practice. The second part serves as a theoretical framework, introducing the concepts of forgetting and forgiveness according to Paul Ricœur, as well as explaining his idea of forgetting as *kept in reserve* and his notion of *difficult forgiveness*. In the third part, the effectiveness of a right to be forgotten in personnel decisions will be discussed. Following this discussion, we will propose an approach towards a responsible HRM practice by shedding light on the concept of forgiveness in the workplace and how an attitude of remembering and forgiving fosters a constructive and failure-tolerant organizational climate. In this step, we will also derive practical implications for a Humanistic Management approach to the use of online information in personnel decision-making. The article closes with a concluding reflection on how employers’ forgetting *kept in reserve* and *forgiveness* benefit human flourishing and relationships within an organization.

## The Use of Information from the Web in Human Resource Management

Companies’ use of online information and individuals’ desire to keep personal information on the web private have been subject to tensions in various respects. In an HRM context, it is not new that social media have been used by companies in recruitment, selection, and disciplinary situations (Lam 2016). More generally, companies’ practice of obtaining information about workers from informal and non-institutional online sources in order to make personnel decisions is referred to as “cybervetting” (Berkelaar 2014). In recruitment and selection, cybervetting is often used as a social media background check or as a source of information in addition to the résumé (Berkelaar 2017). Thereby, a mixture of sources, such as search engines, social media sites, aggregators, e-commerce, virtual worlds, or (micro-)blogs are consulted (Berkelaar and Buzzanell 2014; Berkelaar 2017). These additional sources of information from the web can assist employers in verifying application documents (Brown and Vaughn 2011) or drawing conclusions on applicants’ future job performance (Roth et al. 2016) at low costs (Clark and Roberts 2010; Brown and Vaughn 2011; Chauhan et al. 2013; Jeske and Shultz 2016). In particular, information from social media sites is considered useful in predicting applicants’ personality (Kluemper and Rosen 2009; Rosen et al. 2018) and assessing their potential fit with an organization (Becton et al. 2019). Another frequent argument for the use of social media in selection is to avoid the risk of irresponsible or even criminal employee behaviour, thus protecting the company’s stakeholders from harm (Kluemper 2013). Organizations can even be sued for negligent hiring if they knowingly hire applicants who cause harm to others (Lam 2016). Against these arguments, applicants can be refused if their social media profiles reveal content like provocative or inappropriate photographs, pictures showing alcohol or illegal drug use, or negative comments on previous employers or colleagues (Brown and Vaughn 2011). However, applicants may also be turned down based on demographic information, such as race, gender, age, or disability status, which leads to discrimination against protected groups (Kluemper and Rosen 2009; Brown and Vaughn 2011). By disclosing such information to potential employers, social media sites give answers to questions that are often not allowed to be asked during a job interview (Jones and Behling 2010). Apart from potential discrimination against protected groups, online information on candidates may as well be untrue, incomplete, or taken out of context (Slovensky and Ross 2012; Lam 2016), which questions the accuracy of cybervetting. However, the most frequent criticism as to the ethicality of cybervetting is that employers’ access to private social media activities raises privacy concerns (Black et al. 2015).

Similar tensions also arise from the use of online information in monitoring and disciplining current employees (Lam 2016): The aforementioned CareerBuilder survey revealed that a third of employers (34 %) have reprimanded or fired an employee based on content found online (CareerBuilder 2018). From an employer’s perspective, there exist a variety of reasons for monitoring employees’ social media channels (Lam 2016). As in the case of cybervetting applicants to avoid negligent hiring, employers attempt to prevent harmful or even criminal behaviour as part of their duty to ensure a safe and harassment-free working environment. Another reason is to protect the company against leakage of confidential information or damage to its reputation. However, monitoring employees’ social media activities also raises privacy infringement issues (Lam 2016), as well as concerns regarding disruption of traditional employment relations and a shift in public/private boundaries in organizational life (McDonald and Thompson 2016). With respect to the lawfulness of monitoring employees’ electronic communication, legislations around the globe have adopted very different approaches: For example, the Electronic Communications Privacy Act 1986 generally allows US employers to monitor their employees’ social media activities under certain circumstances. Yet, the question remains whether it can be considered an ethical practice, even in situations where social media monitoring is not prohibited by law (Lam 2016). Because it allows employers to access their employees’ online posts even outside the working sphere (Lam 2016; McDonald and Thompson 2016), employee monitoring has the potential of shifting the power balance in the employment relationship towards employers (Lam 2016).

While critics regard online checks on both, applicants (Clark and Roberts 2010; Stoughton et al. 2015) and current employees (Lam 2016; McDonald and Thompson 2016) as transgressions of boundaries between personal and professional space, laws and regulations to control the use of online media seem to lag behind: Kwak et al. (2021) refer to a gap between ethical issues as part of technological advances, such as invasion of privacy in a cybervetting context, and regulations to address these issues. As a result of access to personal information on the web by unknown third parties, many internet users started expressing the desire to have unpleasant information about themselves removed (Kim and Kim 2017), or, in other words, to be “forgotten” from the web (Mayer-Schönberger and Cukier 2013). In the following sections, we will argue that a ruling like the right to be forgotten can only provide an illusion of protection against unpleasant memories on the web, and that it cannot guarantee a complete “forgetting” of past mistakes. We will begin our argumentation by presenting the notions of forgetting and forgiveness according to French hermeneutic philosopher Paul Ricœur.

## A Ricœurian Approach Towards Forgetting and Forgiving

Scholars from a variety of disciplines agree that remembering and forgetting are key activities for us as human beings. According to Weber (1946), without memory of the past, we fail to appreciate the consequences of our actions, both good and evil. By archiving past experience and providing orientation for future conduct, memory is thus inextricably linked with personal identity (Sison 2016). However, the literature suggests that the ability to forget represents an equally important ingredient of a humane social order. Forgetting has for long been subject to observance and research by scientists, historians, politicians, philosophers, writers and poets. Rather than representing some unfortunate flaw of the human brain, forgetting constitutes an important feature that filters the sensory flood and supports human mental activity (Bannon 2006). Already the German philosopher Friedrich Nietzsche, in his essay *On the utility and liability of history for life*, emphasizes the importance of the human ability to forget for achieving happiness (Nietzsche 1998).

It is therefore that we turn to French hermeneutic philosopher Paul Ricœur (1913–2005) to demonstrate that human forgetting is more than the mere absence of memory. The method of hermeneutics is derived from the Greek words *hermeneuein*, *hermeneia*, and *hermeneus* (Seebohm 2007) and refers to practices of interpretation and theories of understanding, as well as associated fields of translation, communication, and information exchange (Dyer 2010). As Ricœur’s hermeneutics approach takes an enduring interest in the analysis of human action and an understanding of the human condition (Reagan 1998), it has been referred to in a number of management and organization research contexts. For example, Dion (2017) turns to Ricœur’s philosophy to assess the narrativity of corporate citizenship, social responsibility and sustainability reports. Hummels et al. (2021) refer to Ricœur to demonstrate that the grammar of love is crucial in responsible organizations. A recent publication by Espedal and Carlsen (2021) on the role of sacred stories in faith-based health care organizations draws on Ricœur’s ethical philosophy to examine the role of the sacred in organizational values work. Ricœur’s particular interest in “bringing to bear the methods of contemporary language analysis and hermeneutics on the question of the meaning of religious language” (Reagan 1998, p. 41) guides an argumentation towards a practical wisdom approach that employs religious and spiritual traditions as a point of reference for responsible management practice in the article by Habisch and Bachmann (2016). In the study by Deslandes (2012) on Ricœur’s perspectives on the possibility of ethics in institutions in a practical wisdom context, it becomes very clear how Ricœur opposes a vertical and mechanistic conception of management. Rather, management is to be understood as a social construction (Deslandes 2012) – a view that is particularly relevant again in the literature on Organizational Memory Studies, which is concerned with the question of how organizations store information (Fiedler and Welpe 2010). In this context, authors like Rowlinson et al. (2010) cite Ricœur (2004) to demonstrate that remembering in organizations is a social activity rather than just a resource that can be stored and retrieved, as conceptualized in a purely mechanistic model of organizational memory. It is particularly Ricœur’s understanding of remembering and forgetting, which will be of interest for the discussion in this article, as it opposes a mechanistic and binary understanding of the two concepts. In our endeavour to propose an approach towards a responsible HRM practice that fosters human flourishing and personal growth by learning from past mistakes, we also review Ricœur’s perspective on forgiveness in this chapter. Thereby, his book *Memory, history, forgetting* (Ricœur 2004) serves as a basis for the discussion of how unpleasant online memories can be dealt with in an HRM context.

### Forgetting Kept in Reserve

In *Memory, history, forgetting*, Ricœur (2004) points to the dilemma of the duality of forgetting and remembering. On the one hand, the admonition not to forget appears to be universal in a number of contexts, such as in the writings of Herodotus, which aim to preserve the glory of the Greeks and the Barbarians for posterity. But at the same time, humans are intimidated by the thought of a memory that would never forget anything (Ricœur 2004). Consequently, he dismisses any antagonism of forgetting in favour of a more comprehensive approach, which takes the profundity of forgetting into account. A similar thought was introduced by Bannon (2006), who regards the duality of forgetting and remembering as an unnecessary limitation of options, especially in a world in which technology provides a breadth of useful tools and solutions for human society. Noting that forgetting could “no longer be in every respect an enemy of memory” (Ricœur 2004, p. 413), Ricœur proposes to search for the right measure between memory and forgetting. He thus stresses that in essence, forgetting is not a problem of if or if not, but one of *more or less*. In particular, Ricœur (2004) distinguishes two forms of forgetting:

- profound forgetting as forgetting through the erasing of traces, and
- forgetting kept in reserve (*oubli de réserve*), which can be understood as a backup forgetting.

Concerning the latter concept, Ricœur (2004) considers forgetting in relationship to the notion of latency: A memory never disappears completely, but is kept in the back of people’s minds and can suddenly reappear. In this sense, above all, forgetting consists in putting memory in reserve rather than erasing it, whereby the memory keeps existing in the unconscious. Moreover, forgetting kept in reserve is conceived of in reference to its psychic trace. Ricœur gives the example of a happy memory that can reappear as an image coming back to a person by recognizing a good friend. Thus, the traces of memories never find themselves completely erased from the human brain. Rather, their being kept in reserve represents a precondition for a sudden reappearance, and for their subsequent integration into a comprehensive biographical horizon, as well as for the process of forming values and creating wisdom in human beings. Correspondingly, on a collective level, the same dynamic of forgetting as keeping memory in reserve (for example, in historical archives or libraries) also represents the precondition for historical studies in a changed socio-economic environment, as well as their systematic integration into alternative and culturally creative interpretations and representations. As it is memory that allows forgiveness to slow the progress of oblivion, Ricœur’s (2004) notion of forgiveness directly arises from the reflection on time, as explained by Faldetta (2021). In our endeavour to offer an approach towards personnel decision-making based on forgiveness, we proceed by briefly reviewing Ricœur’s (2004) concepts of easy and difficult forgiveness.

### Easy and Difficult Forgiveness

Ricœur distinguishes between easy and difficult forgiveness, whereby easy forgiveness is often encountered in the workplace, as it is characterized by status and power differences. Here, easy forgiveness frequently requires apologies or amends or some other form of punishment in order to symbolically restore the law and the offender as a member of society (Ricœur 2004; Faldetta 2021). Thus, this kind of forgiveness is conditional. However, Ricœur (2004) also refers to a difficult, or unconditional, forgiveness, whereby the difficulty of the forgiveness lies in the disproportion of the depth of the guilt and the height of forgiveness. Here, the Ricœurian notion of forgiveness as a gift conceptualizes forgiveness as an opportunity for a re-start or a rebirth of the guilty: Through acknowledging and reinterpreting memory rather than attempting to cancel the irreversible mistake, the gift of forgiveness weakens the effect of the wrongdoing on present and future. The memory of an offence is thus reinterpreted rather than erased. Thereby, Ricœur stresses that the evil of an action must be separated from the offender for forgiveness to break the bond that ties the offender to the wrongdoing (Ricœur 2004; Faldetta 2021). Therefore, we conclude that difficult forgiveness keeps the memory of an offence in reserve and reinterprets it instead of erasing its traces – a feature that stands in contrast to a right to be forgotten which aims at erasing mistakes from the web. In the following paragraphs, we will demonstrate that the right to be forgotten thus cannot provide an adequate approach towards handling past mistakes in an employment context.

## A Critical Discussion of the Right to be Forgotten

Internet users’ desire to have unpleasant information about themselves removed from online records gave rise to the so-called “right to be forgotten”, which was then established by the European Court of Justice (ECJ) (Kim and Kim 2017). Article 17 of the European General Data Protection Regulation (GDPR) thus grants EU citizens “the right to obtain from the controller the erasure of personal data concerning him or her without undue delay and the controller shall have the obligation to erase personal data without undue delay” (European Parliament 2016, p. 43). The right, as we find it nowadays in the European jurisdiction, has its intellectual roots in the much older French droit à l’oubli, which grants a convicted criminal the right to oppose the publication of his or her criminal history after serving the criminal sentence time (Rosen 2012). Thus, it prevented others from communicating the person’s association with his or her criminal past based on privacy as a human/fundamental right and human dignity (Ambrose and Ausloos 2013). In this context, Ambrose and Ausloos (2013) refer to the case of the murder of the German actor Walter Sedlmayr in 1990. Both perpetrators were convicted, but due to their victim’s fame, the case received considerable public attention and was documented in a Wikipedia entry. After the murderers’ release, a letter was sent to Wikipedia with the request to remove the name of one of the perpetrators from the website, arguing that it invaded their right to privacy under German law. In 2009, the French Secretary of State and Prospective Development of the Digital Economy demanded a droit à l’oubli in the online environment, as well (Ambrose and Ausloos 2013). As a means of defending users’ privacy rights and information autonomy, the present right to be forgotten has received considerable praise (Lindsköld 2018), since it may allow users to erase personal information from the past that affects their present. However, authors like Garcia-Murillo and MacInnes (2017) argue that the right to be forgotten is an inadequate approach to addressing the harm caused to people’s present through personal information from the past for various reasons. The following paragraphs, therefore, summarize the key points of criticism on the right to be forgotten raised in the literature.

### A Conflict of Fundamental Human Rights

Despite all praise for defending individual privacy, the right to be forgotten has been subject to vivid discussions on conflicts between the values of privacy and freedom of speech. Thereby, critics argue that the removal of links under the right to be forgotten reduces the information about a person available to others and thus weakens freedom of speech (Kim and Kim 2017). For example, Jeffrey Rosen, Professor of Law at George Washington University, referred to the right to be forgotten as “the biggest threat to free speech on the Internet in the coming decade” (Rosen 2012, p. 88). A number of scholars claim that, if not balanced properly against the right to free speech, the right to be forgotten may lead to a far less open internet (Rosen 2012; Antani 2015). This effect may come at the expense of the public, because generally accessible pieces of knowledge can be lost – a negative social consequence whose long-term results are difficult to assess (Ambrose and Ausloos 2013).

In this context, the sharpness of the criticism of the right to be forgotten can partly be explained by international differences in the importance of privacy and free speech, which varies from jurisdiction to jurisdiction. For example, common-law jurisdictions like the United States and the United Kingdom place greater importance on free expression, whereas in civil-law jurisdictions like France, Germany, Italy, or Switzerland, privacy concerns prevail (De Baets 2016). This explains why the ECJ as a court dominated by judges from civil-law traditions from Continental Europe appears to put more emphasis on privacy rights than on free speech rights (Kelly and Satola 2017). From that perspective, different sentiments towards the right to be forgotten in Europe and the United States can be explained by a different interpretation of privacy policy on a continuum between dignity and liberty: In Europe, privacy laws are rooted in a deep respect for human dignity and the protection of individuals’ public image (Walker 2012). Accordingly, many Continental European countries perceive the ECJ ruling as a consequence of basic personality rights, encompassing several elements such as dignity, honour, and the right to privacy (Weber 2011). In contrast, in the United States, privacy is generally interpreted in terms of liberty and aims at protecting citizens against governmental intrusions – a distrust in centralized power, which manifests itself in the Fourth Amendment (Walker 2012). It is therefore not surprising that objections against the right to be forgotten were particularly voiced by Anglo-Saxon scholars and commentators, especially from the US (Bode and Jones 2018). Here, freedom of expression, freedom of the press, and a “right to know” have been postulated as the most “fundamental American values” (George 2018, p. 909).

A related issue is the reliance on data controllers in assessing and implementing take-down requests under the right to be forgotten. As the right shifted more responsibilities towards data controllers, search engine operators like Google were the first to operationalize it. However, the process of evaluating link delisting requests places a burden on search engine operators, since the implementation of the right to be forgotten lacks clearly specified guidelines for application within individual member states (Kwak et al. 2021). Furthermore, a number of authors express concerns as to whether private companies like Google are suitable for balancing fundamental human rights like the right to privacy and the right to free speech on the basis of their lack of expertise and a tendency towards selfish economic interests (Abril and Lipton 2014; Bougiakiotis 2016). Similarly, Lindsköld (2018) notes that a private organization cannot be expected to comply with the same standards of transparency as a government institution. While the balancing of the right to privacy and the right to free speech is intensely debated, Hoffman et al. (2016), as well as Uncular (2019) state that link removals under the right to be forgotten do not automatically cause unfavourable memories on the web to be truly forgotten. We will concentrate on the practical and technical implications of this aspect in the next paragraph.

### Practical and Technical Limitations of the Right to be Forgotten

Research has shown that due to practical and technical limitations, the right to be forgotten is difficult to enforce, as personal information hardly disappears completely from the web. In particular, data privacy advocates have criticized the right to be forgotten for being too provisional and leaving too much room for interpretation within individual member states, jurisdictions and cultural areas (Politou et al. 2018). Authors like Antani (2015) note that the right lacks specific criteria or standards by which it can be enacted in practice, as highlighted in the previous discussion on the implementation of the right by search engine operators. For instance, the definitions of “data subject” and “personal data”, as established in Article 17 of the GDPR, are rather broad, making it difficult to clearly identify whose rights should be respected. If the data subject is part of a group (like a photograph of a school class or a family), it remains unclear whose rights need to be honoured in case one person has issued a take-down request and the others have not (Shoor 2014). Therefore, an employer who is truly interested in candidate information delisted under the right to be forgotten would quickly discover the required information by searching for other group participants’ names. For example, by using related search terms other than the candidate’s name, an employer may discover any news articles or websites related to a candidate, even if these sites are subject to the right to be forgotten (Antani 2015; Hoffman et al. 2016; Lindsköld 2018). As a result, the right to be forgotten does not guarantee an actual forgetting of any information (Hoffman et al. 2016), but merely makes it more difficult to find. While the viability of the right to be forgotten is therefore already questionable from a technical and practical perspective, in the following sections we will question the effectiveness of such a ruling when applied in the workplace.

### The Right to be Forgotten in Employment Relationships

As the right to be forgotten involves the removal of links from search results (Kim and Kim 2017), it clearly refers to a concept of forgetting through the erasing of traces according to Ricœur (2004). However, with Ricœur (2004), we have shown that forgetting is not binary, but a matter of more or less, deeming the complete erasure of a memory impossible. Moreover, memories on the web are collective in nature. In this context, Garcia-Murillo and MacInnes (2017) refer to the leakage problem in relation to the right to be forgotten: Thereby, personal information on the web may leak far beyond the intended audience through movement across different platforms, being viewed, shared, and copied by different people. While the right requires others to forget (De Baets 2016), this wide range of third-party (“secondary”) uses makes it practically impossible to delete a piece of information completely (Ausloos 2012). Thus, when people post content they regret later, the act of removing the information may draw even more attention to the issue (Lampinen et al. 2011; Garcia-Murillo and MacInnes 2017). In this case, a so-called “Streisand effect” (Xue et al. 2016) may occur when the impact of an attempt to repress information becomes even more negative. The term was coined by blogger Mike Masnick (2005) for describing cases of legal overreach by trademark and copyright holders or, more generally, situations in which a piece of information intended to be hidden or removed gets spread more widely on the web (Xue et al. 2016). It originates from celebrity Barbra Streisand’s suing a photographer for invasion of privacy, because he uploaded a photo showing Streisand’s Malibu mansion on a publicly accessible online photo database. Without Streisand’s lawsuit, few people would have even noticed the existence of the photo. Through her attempt of having the photo removed, however, the case ended up gaining enormous public attention (Jansen and Martin 2015). With Ricœur, we thus have to admit that for reaching a perfect forgetting, it is necessary not only to completely erase what we want to forget, but also to prevent any future attempt to remember, since the results of such an attempt would never be controllable. But what are the consequences of future attempts to remember? In a liberal society, it seems difficult to ban the mere search for information. A more comprehensive right to erasure of personal data on the web, however, executed in the form of a prohibition of even the attempt to remember, would necessarily address a multitude of agents of very different size, status, and nature: It would mean addressing society as a whole, which seems impossible. We therefore have to acknowledge that any right which obliges other people to forget remains very difficult to enforce.

For applicants who attempt to increase their chances of being hired through the delisting of links to unflattering information from the web, this means that the right to be forgotten can only remain an illusion of protection. More precisely, it can never provide any protection from the memory itself, but will leave employees with a sword of Damocles over their heads: Even if a confrontation with the unpleasant memory was successfully avoided at the time of recruitment, it may reappear occasionally later on, creating an even more tormenting situation. In the worst case, the “Streisand effect” (Xue et al. 2016) occurs, forcing candidates to explain why they failed to mention a certain matter in the first place. In this respect, the erasure of traces on the web may even turn into a trap, as it leads applicants to believe the undesired memory is truly forgotten. Consequently, even already well-integrated employees may find their job position hampered by the futile attempt to hide something unpleasant. We must therefore ask whether the mere existence of past events really says anything about the engagement, creativity or seriousness of the person as a job candidate: Someone pictured drunk on a party may still be a good employee, irrespective of what he or she does beyond working hours (Chauhan et al. 2013) and may not necessarily be an alcoholic. Similarly, not everyone holding a hunting rifle in a picture is a mass murderer (Elzweig and Peeples 2009). Rather, much more revealing than “bruta facta” is the manner in which those unpleasant memories are kept in reserve and find themselves integrated in a personal learning history. In the next section, we will thus propose an approach towards a responsible handling of unpleasant memories on the web. We will show how a manager’s attitude of forgiveness towards existing and prospective employees’ mistakes, as well as an appreciation for their learning history can foster a constructive corporate culture in the long run.

## A Responsible Handling of Unpleasant Memories on the Web

### Forgiveness and Forgetting

Since responsible recruitment, selection, and disciplinary processes should ultimately benefit the relationship between members of an organization, it is worthwhile taking a closer look at the concept of forgiveness and the role of remembering in HRM decision-making. As argued by Feldman and Feldman (2006), remembering is key in creating goodness in relationships within organizations, because it enables the identification of goodness and caring about the organization and its members. Thereby, forgiveness and gratitude play an important role, as they represent backward-looking emotions, which need remembering (Margalit 2004; Feldman and Feldman 2006). Forgiveness not only represents an important trait for today’s managers, but it can also be a powerful element of an overall HRM strategy (Kurzynski 1998). Yet, it is important to note that real forgiveness is distinct from forgetting (Cameron and Caza 2002; Margalit 2004). To illustrate the differentiation between true forgiveness and forgetting, Margalit (2004) uses the metaphor of writing a text: When people are dissatisfied with something they have written, they can either get rid of it by deleting it, or by crossing it out. While deleting implies total erasure of what has been written, crossing out leaves traces of the error behind, which allows the writer to learn from it. Therefore, for achieving true forgiveness, it is more preferable to disregard a sin than to forget it (Margalit 2004). Following this logic, a manager is not asked to simply condone a wrongful act (Kurzynski 1998), or ignore his or her disapproval of certain actions, but to evaluate a person beyond the mere negative behaviour and look at that person as still *good enough*, despite past mistakes. As Ricœur (2004) teaches us, forgiveness separates the person from his or her wrongful past and thus requires managers to see an applicant or employee in a new, more favourable light (Murphy and Hampton 1988). Ricœur’s (2004) difficult forgiveness does not cancel the mistake of the perpetrator, but represents an opportunity for a re-start by reinterpreting the memory and safeguarding it for the future (Faldetta 2021). It is therefore a memory kept in reserve, which allows this kind of forgiveness. A famous example of such a re-start is the biblical story of the conversion of St. Paul in the New Testament (Bible 2019, *Acts* 9:1–9:8). The Pharisee Saul brutally persecuted the followers of Jesus until he had a vision of Jesus appearing to him on his way to Damascus. Following this revelation experience, Saul ceased to persecute the early Christians and became a convinced disciple of Jesus, calling himself Paul. For the rest of his life, he preached the word of Jesus and told the people about his conversion and his wrongful past. Not only does the story imply that even the greatest sinner can attain Divine Grace. With his conversion, the wrongdoer Saul is not only forgiven and regarded as still *good enough*, despite his wrongful past. Rather, his ability to see his past in a new light and talk about it represents a special quality that makes him even more convincing and trustworthy, even among his former enemies. We must therefore conclude that not an erasure of traces is the precondition for forgiving and moving beyond past mistakes. Rather, it is the act of keeping a mistake in reserve and changing the relationship with its memory (Ricœur 2004; Faldetta 2021), which allows a manager to truly forgive applicants and employees by accepting them despite, or even *because* of past mistakes. In the next paragraph, we show how the workplace situation, as well as economic considerations, affect the willingness and ability of organizational members to practice unconditional, or in Ricœur’s (2004) terms, *difficult* forgiveness.

### Easy and Difficult Forgiveness in the Workplace

When applying Ricœur’s concept of forgiveness in an organizational context, it has to be acknowledged that the practice of forgiveness is constrained to a great extent by workplace organization (Stone 2002; Barclay and Saldanha 2016). Forgiveness usually occurs in close relationships with family, friends, or spouses, which are characterized by commitment, pro-relationship behaviour, and trust (Wieselquist et al. 1999; Bies et al. 2016). These characteristics are rarely found in professional relationships, since in workplace contexts, the parties involved are usually organized in arrangements that are aimed at achieving organizational goals (Aquino et al. 2003; Barclay and Saldanha 2016) and typically perform tasks working together with colleagues and supervisors by assignment rather than by choice. Thus, in such an environment, there is more at stake than the preservation of relationships, as the achievement of economic and career goals may also depend on conflict resolution in the workplace (Aquino et al. 2003; Thompson and Korsgaard 2019). While forgiveness can indeed result from a true internal change towards the transgressor, it is likely that in a professional environment, it occurs out of self-serving motivations (Baumeister et al. 1998; Zheng and Dijke 2020). Since most business firms qualify as what Bies et al. (2016) refer to as utilitarian organizations, a manager’s decision to forgive may be based on a cost-benefit analysis (see McCullough et al. 2013). Research by Burnette et al. (2012) shows that forgiveness most likely occurs when the transgressor has high relationship value and low exploitation risk – a finding that Bies et al. (2016) discuss with respect to its consistency with interdependence (Kelley 1979) of the parties involved. It is therefore to conclude that a manager’s forgiveness may indeed occur as the result of a cost-benefit analysis, because he or she expects a certain value in exchange, or may feel dependent on the transgressor’s performance.

As a manager’s own professional career is often linked to organizational goals, a manager’s forgiveness of an employee may also be motivated by the desire to improve overall performance or decrease the turnover of highly qualified staff. Indeed, a number of researchers found forgiveness to contribute to a number of positive outcomes, such as employee wellbeing, satisfaction, and retention (see Aquino et al. 2003; Caldwell and Dixon 2010; Fehr et al. 2010; Thompson and Korsgaard 2019). Furthermore, authors like Stone (2002) show that the costs of not forgiving can negatively impact organizational performance to a great extent, leading to ineffective job performance and employee turnover. An organization’s ability to identify, manage, position, and *retain* talent is decisive in achieving long-term organizational sustainability and success, especially in the face of economic crises (McDonnell 2011). Against this background, managers may forgive their high-performing employees out of their own interest, because they find that organizational goals are at stake, which are ultimately tied to their own managerial careers. As discussed by Faldetta (2021), such an approach to forgiveness would be characterized more in a transactional sense, as an exchange or a negotiation (Miceli and Castelfranchi 2011). This kind of forgiveness would qualify as conditional, or what Ricœur (2004) would consider *easy* forgiveness, as it is conditioned by status and power differences. However, Faldetta (2021) highlights the importance of both, conditional (easy) and unconditional (difficult) forgiveness in the workplace, whereby unconditional forgiveness implies that a manager forgives a mistake purely as an act of caring for the needs of others (Garrard and McNaughton 2003; Faldetta 2021). In the following paragraphs, we intend to show that a culture of love, forgiveness, and trust can contribute to the development and flourishing of organizational members, as well as harmonious cooperation in achieving a common good. We will conclude that such an approach towards remembering and forgiveness in organizations is consistent with Humanistic Management (Melé 2016) in several ways.

### A Culture of Love, Forgiveness and Trust as a Humanistic Management Approach to HRM

As noted by Faldetta (2021), organizational members who practice unconditional forgiveness contribute to a culture of love, forgiveness, and trust, which has the ability to empower individuals’ capabilities. In such a working environment, forgiving mistakes as they happen can help create a positive culture for risk-taking and creativity that allows organizations and individuals to achieve their potential (Lennick and Kiel 2008). Consequently, a failure-tolerant organizational culture is created, based on “organizational values, norms, and artifacts that imply that failures are constructively handled, openly addressed, and freely communicated; that the causes and underlying mechanisms of failures are analyzed for improvement; and that failures are even actively encouraged” (Vomberg et al. 2020, p. 120). In the entrepreneurship literature, failure has often been viewed as an opportunity for learning (Yamakawa and Cardon 2015). Hence, previously unsuccessful founders are often better able to obtain venture capital funding than novices (Nahata 2019). An organizational culture that not only tolerates, but *welcomes* failure as an opportunity for learning, can be nourished by symbolic acts like an award (Van Maanen and Schein 1977). However, it is also implicitly influenced by group observation (Harmeling et al. 2017) in employees’ daily interactions with mentors and coworkers (Lam et al. 2010). This in particular highlights the importance of the hiring process as the first source of information on the corporate culture for applicants and new employees (Gilliland 1993), but also the role of managers in their dealing with employees’ mistakes, thereby strongly influencing the perception of the corporate culture (Naumann and Bennett 2000; Fehr and Gelfand 2012). In this case, the forgiveness of mistakes would not be limited to content that managers discover in web searches. Rather, it would be found deeply rooted in a failure-tolerant culture in the workplace, which is driven by the members of the organization, where the managers act as role models. The reliance on a right to be forgotten, in contrast, contributes to a judgmental society, in which individuals feel pressured to present a reputable public image (Garcia-Murillo and MacInnes 2017).

An approach to unpleasant memories on the web based on remembering and forgiveness is consistent with Humanistic Management in several respects. In his 2016 article, Melé presents a set of seven propositions that characterize humanism: (1) wholeness, (2) comprehensive knowledge, (3) human dignity, (4) development, (5) common good, (6) transcendence, and (7) stewardship-sustainability. Our approach first and foremost corresponds to Melé’s (2016) fourth proposition, according to which humanism calls on human beings to develop and flourish. This includes not only biological, but also intellectual growth, whereby humans continuously learn from others through reflecting and acting (Melé 2016). By remembering, acknowledging, and openly discussing applicants’ past mistakes instead of ignoring them, or even dismissing applicants without further notice, a manager allows applicants, as well as existing employees to learn from such mistakes and fosters their development in the sense of reflecting and acting. Furthermore, according to Melé’s (2016) fifth proposition, humanism emphasizes relationability and sociability, as well as harmonious cooperation to achieve a common good. By creating goodness in the relationships within an organization through remembering and forgiveness, which ultimately means caring about others (Feldman and Feldman 2006), our approach complies with human sociability, understood as a “natural tendency to live in society and to establish bonds between the person and the social groups to whom we belong” (Melé 2016, p. 43). Through exercising unconditional, or, in Ricœur’s terms, *difficult* forgiveness, a manager moves beyond resentment not out of superiority, but with a focus on caring for the needs and concerns of other members of the organization (Garrard and McNaughton 2003; Faldetta 2021). Moreover, an open discourse on applicants’ unpleasant memories on the web responds to human relationability through dialogue (Melé 2016). Taking humans’ social nature and their need for personal growth as starting points, one possible approach to Humanistic Management is centred around the adoption of an organizational culture which fosters character and develops people (Melé 2003). In addition to forgiveness of mistakes in existing employment relationships, a responsible hiring process characterized by remembering and forgiving would thus support the formation of such constructive organizational cultures, as well as harmonious and constructive relationships within organizations. This can, however, only be achieved by paying close attention to candidates’ positive traits and by closely engaging with them in the hiring process. Thereby, it is crucial to consider a candidate’s personal learning history, as well as the distance between the person at the time of applying for the job and the piece of information discovered on the web. Turning back to an earlier work of Ricœur (1974), we need to acknowledge that a written text (like a piece of information on the web) is different from the spoken word, as it becomes independent from the speaker so that its meaning is no longer identical with the intended meaning of the communicator: The text thus becomes subject to a re-interpretation in the contemporary context of the reader (Habisch and Bachmann 2016). In the case of the discovery of unpleasant online memories in an employment context, the information found on the web can be interpreted in a completely different way than it was intended when it was posted. In the meantime, the applicant or employee may have experienced significant personal development, which makes it necessary to interpret the online information in the contemporary context of that person, acknowledging his or her development and personal growth. In the following paragraphs, we will formulate implications and recommendations for practitioners aimed at establishing processes of personnel decision-making that acknowledge and forgive individuals’ past mistakes and make responsible use of information from web searches.

### Practical Implications and Recommendations

Based on the definition of a failure-tolerant organizational culture by Vomberg et al. (2020), open discourse on failure that allows people to learn from mistakes should be encouraged in organizations’ everyday operations. Through leading by example and exercising forgiveness, managers and leaders can actively shape a climate of forgiveness in their daily actions (see Naumann and Bennett 2000; Fehr and Gelfand 2012). Thus, even an applicant’s inappropriate comment in the employment interview or an employee’s mistake in delivering a service can be kept in reserve without overrating it and without losing sight of the person’s positive qualities. This can, however, only be done through close engagement with applicants and current employees. Unfortunately, cybervetting has been found to be an “extractive” rather than an “interactive” search for information, as it seldom involves two-way communication with applicants (Berkelaar 2014, 2017; Berkelaar and Buzzanell 2014), leaving them without a chance to comment on online information directly. Therefore, to avoid the exclusion of potentially gifted candidates, it is recommended that organizations provide the opportunity for two-way communication already during the selection process (Hurrell et al. 2017), thereby allowing candidates to comment on past behaviour. As Ricœur (1974) teaches us that a piece of information discovered on the web becomes subject to a re-interpretation in the contemporary context of the reader (Habisch and Bachmann 2016), it should thus “invite” employer and (potential) employee to start a dialogue and make sense of that information in the contemporary world. This can be done, for example, if a manager sits down with the person in an employment interview or an employee appraisal, and asks him or her to take a position on the information from the web.

Although we support a selection process that makes informed decisions based on online information, we recommend that organizations should not lose sight of the ethical risks associated with the use of online media in personnel decisions, such as discrimination (Kluemper and Rosen 2009; Jones and Behling 2010; Brown and Vaughn 2011), privacy concerns (Black et al. 2015), or the reliance on inaccurate or even false information (Slovensky and Ross 2012; Lam 2016). To address these issues and design fair, responsible, and informed online search processes, Vosen (2021) provides three key recommendations for employers: The first is to standardize the cybervetting process in order to avoid differential treatment of candidates in the selection process. Moreover, clear responsibilities should be assigned to the people who are involved in the selection process (Vosen 2021). For example, while it is recommended to have the HR department perform the cybervetting, as they are more familiar with issues of validity, adverse impact, and disparate treatment than practising managers, companies can hire third-party consultants to screen applicants on their behalf. Such service providers screen social media profiles for a variety of characteristics and present a report that excludes demographic information in order to prevent discrimination (Davison et al. 2016). The second key recommendation is to ensure that the information obtained from the web is relevant for the position that needs to be filled, which can be achieved through a job analysis prior to the selection process (Vosen 2021). The very least a manager can do in the hiring process is to consult traditional selection methods equally (Landers and Schmidt 2016) and to perform cybervetting late in the process (Davison et al. 2016) when basic traits and qualifications are already known. Thirdly, as part of the general recommendation for organizations to provide an adequate degree of transparency and two-way communication with applicants (Vosen 2021), a number of authors also discuss the possibility of informing job candidates about the cybervetting process beforehand (Slovensky and Ross 2012; Hoek et al. 2016; Lam 2016). This way, hiring managers may avoid the emergence of distrust in the employer-employee relationship from the very beginning. Since a culture of forgiveness and failure tolerance can be nourished by symbolic acts (Van Maanen and Schein 1977), the organization’s attitude towards failure could be written down in a document, like a code of conduct. Such a document could also include guidelines for responsible use of information technology in recruitment, selection, and disciplinary actions (see Lam 2016).

While we advocate for an approach to HRM decision-making based on forgiveness, two limitations should be noted here. Although we argue that a responsible selection process based on remembering and forgiveness should be established to make the reliance on a policy like the right to be forgotten unnecessary in an employment context, there are extreme cases that should be addressed through adequate rules and regulations (Kwak et al. 2021): Comments on people’s social media profiles or tagged photos may be published without a person’s consent (Chauhan et al. 2013). Users may also become victims of identity theft (Connerley et al. 2001; Davison et al. 2012) or libel if false and defamatory information is posted on the web (Davison et al. 2012). The second limitation concerns the extent to which some managers should forgive applicants and employees. This is particularly true for companies that bear an increased responsibility towards their stakeholders when making personnel decisions in order to reduce the risk of irresponsible or even criminal behaviour (Kluemper 2013). This may apply, for example, when organizations are involved in public safety, such as trucking, child care, or public sector activities (Slovensky and Ross 2012). Such companies may have an increased interest in avoiding negligent hiring lawsuits through the use of online background checks before hiring a person (Lam 2016) and may therefore only have limited capacity to forgive past mistakes.

## Conclusion

As cybervetting increasingly becomes common practice in hiring and digital tools enable employers to monitor employees’ social media activities, the right to be forgotten addresses users’ need to have unpleasant memories removed from the web. In this article, we have shown that such a right does not represent an appropriate solution to handle unpleasant memories on the web. Instead, it can only provide an illusion of protection for applicants and employees, because it is impossible to enforce a complete erasure of traces. With Ricœur (2004), we have demonstrated that memories are not binary, but a matter of more or less. In an environment in which information posted on the web gets shared and copied across different platforms by a variety of people, it is impossible to guarantee a complete erasure of traces from the web and from people’s minds, neither can law-making effectively forbid anyone the mere search for information. If unwanted information is retrieved during the search process, or if employees are later confronted with the behaviours they wanted to conceal during the selection process, this may lead to even worse consequences. As a result, such an attempt to protect applicants’ privacy may end up in a distortion of the employee-employer relationship, as it establishes a climate of distrust from the very beginning.

To account for a responsible handling of unpleasant memories on the web consistent with Humanistic Management, a manager’s attitude of forgiveness and tolerance of mistakes can thus be regarded as more beneficial for human flourishing within an organizational culture of harmonious cooperation in the long run. According to Ricœur’s (2004) notion of forgetting as “kept in reserve”, the adequate challenge is not to irretrievably silence unpleasant memories of past mistakes through a right to be forgotten. Rather, a remembering of mistakes should be possible in order to truly forgive and see existing and prospective employees’ positive qualities beyond, or even *because* of their mistakes. By practising what Ricœur (2004) considers difficult forgiveness, an organization can cultivate a constructive climate, which empowers the capabilities of its members through learning from past mistakes. Employment relationships would thus benefit to a great extent if existing and prospective employees were given the possibility to position themselves on any negative information on the web in the context of their personal learning history.

## Data Availability

Not applicable.
